# Supplementary material for: Detailed molecular and epigenetic characterization of the pig IPEC-J2 and chicken SL-29 cell lines
Source: iScience. 2023 Feb 20;26(3):106252. doi: 10.1016/j.isci.2023.106252 (PMC10018572; doi:10.1016/j.isci.2023.106252)
Supplement: Data S2. Complete homer output for identified motifs in Chicken SL-29, related to Tables 5 and 6 — Homer motif analysis results for histone modifications H3K4me1, H3K4me3, H3K27ac, enhancers, and ATAC-seq of chicken SL-29 cell line. Parameters for possible false positives is as mentioned earlier for S5. [file mmc3.zip › Data_S2/S6/Chicken_SL_29/peak_files_CTCF/knownResults.html]

peak\_files\_CTCF - Homer Known Motif Enrichment Results


# Homer Known Motif Enrichment Results (peak\_files\_CTCF)

Homer *de novo* Motif Results  
Gene Ontology Enrichment Results  
Known Motif Enrichment Results (txt file)  
Total Target Sequences = 29475, Total Background Sequences = 29070

|  |  |  |  |  |  |  |  |  |  |  |  |
| --- | --- | --- | --- | --- | --- | --- | --- | --- | --- | --- | --- |
| Rank | Motif | Name | P-value | log P-pvalue | q-value (Benjamini) | # Target Sequences with Motif | % of Targets Sequences with Motif | # Background Sequences with Motif | % of Background Sequences with Motif | Motif File | SVG |
| 1 | A C T G C T A G T C G A C G A T C A T G G C T A A T C G C G A T G T A C G C T A A G C T G T A C | Fra1(bZIP)/BT549-Fra1-ChIP-Seq(GSE46166)/Homer | 1e-1194 | -2.750e+03 | 0.0000 | 5161.0 | 17.51% | 1569.8 | 5.40% | motif file (matrix) | svg |
| 2 | C T A G T C G A A C G T A C T G C G T A A T G C A C G T G T A C C G T A A G C T G A T C G T A C | Atf3(bZIP)/GBM-ATF3-ChIP-Seq(GSE33912)/Homer | 1e-1183 | -2.726e+03 | 0.0000 | 5748.0 | 19.50% | 1910.0 | 6.57% | motif file (matrix) | svg |
| 3 | C A G T T G C A A C G T A C T G C G T A A T C G C G A T T G A C C G T A A C G T | BATF(bZIP)/Th17-BATF-ChIP-Seq(GSE39756)/Homer | 1e-1163 | -2.678e+03 | 0.0000 | 5506.0 | 18.68% | 1794.0 | 6.17% | motif file (matrix) | svg |
| 4 | A C T G C A T G T C G A A C G T A C T G C G T A A T C G A C G T G T A C C G T A G A C T A G T C | Fos(bZIP)/TSC-Fos-ChIP-Seq(GSE110950)/Homer | 1e-1159 | -2.671e+03 | 0.0000 | 5391.0 | 18.29% | 1731.4 | 5.95% | motif file (matrix) | svg |
| 5 | C A T G C T A G T C G A A C G T A C T G C G T A T A G C C G A T T G A C C G T A A G C T G A T C | Fra2(bZIP)/Striatum-Fra2-ChIP-Seq(GSE43429)/Homer | 1e-1155 | -2.660e+03 | 0.0000 | 4544.0 | 15.41% | 1276.5 | 4.39% | motif file (matrix) | svg |
| 6 | C T A G T C G A G C A T C A T G G C T A T A G C C G A T G T A C C T G A A G C T | JunB(bZIP)/DendriticCells-Junb-ChIP-Seq(GSE36099)/Homer | 1e-1132 | -2.608e+03 | 0.0000 | 4875.0 | 16.54% | 1472.7 | 5.06% | motif file (matrix) | svg |
| 7 | T C G A A C G T C A T G G C T A T A G C C G A T G T A C G C T A A C G T A T G C | AP-1(bZIP)/ThioMac-PU.1-ChIP-Seq(GSE21512)/Homer | 1e-1094 | -2.521e+03 | 0.0000 | 6006.0 | 20.37% | 2165.3 | 7.44% | motif file (matrix) | svg |
| 8 | C T A G T C G A C G A T A C T G C G T A T A C G A G C T T G A C G C T A A C G T G A T C T A G C | Fosl2(bZIP)/3T3L1-Fosl2-ChIP-Seq(GSE56872)/Homer | 1e-958 | -2.208e+03 | 0.0000 | 3246.0 | 11.01% | 791.8 | 2.72% | motif file (matrix) | svg |
| 9 | C T A G T C G A A C G T A C T G C G T A T A G C C G A T G T A C C G T A A G C T G A T C G T A C | Jun-AP1(bZIP)/K562-cJun-ChIP-Seq(GSE31477)/Homer | 1e-794 | -1.828e+03 | 0.0000 | 2480.0 | 8.41% | 557.0 | 1.91% | motif file (matrix) | svg |
| 10 | T G C A A G C T C T G A A T C G G A C T C T A G G T A C G A T C G T C A A G T C G T A C G A C T C T A G A T C G G C A T C A T G C A T G G A T C G T A C C T G A | CTCF(Zf)/CD4+-CTCF-ChIP-Seq(Barski\_et\_al.)/Homer | 1e-435 | -1.003e+03 | 0.0000 | 1829.0 | 6.20% | 522.6 | 1.80% | motif file (matrix) | svg |
| 11 | A G T C C T G A A G T C C G A T C A G T G A T C A T G C A C T G A T C G G A C T | Fli1(ETS)/CD8-FLI-ChIP-Seq(GSE20898)/Homer | 1e-421 | -9.707e+02 | 0.0000 | 6813.0 | 23.11% | 3960.1 | 13.61% | motif file (matrix) | svg |
| 12 | C G A T T A C G T G A C G A C T C A T G C G T A T A C G A C G T G T A C C T G A | Bach2(bZIP)/OCILy7-Bach2-ChIP-Seq(GSE44420)/Homer | 1e-403 | -9.287e+02 | 0.0000 | 1710.0 | 5.80% | 491.9 | 1.69% | motif file (matrix) | svg |
| 13 | A T G C A G T C C T G A A G T C C G A T A C G T A G T C A G T C A C G T A T C G G A C T A C G T | Etv2(ETS)/ES-ER71-ChIP-Seq(GSE59402)/Homer | 1e-369 | -8.519e+02 | 0.0000 | 5443.0 | 18.46% | 3037.3 | 10.44% | motif file (matrix) | svg |
| 14 | T C G A T A G C G T C A A C T G A C T G C G T A C G T A C T A G A G C T T C A G | ERG(ETS)/VCaP-ERG-ChIP-Seq(GSE14097)/Homer | 1e-358 | -8.251e+02 | 0.0000 | 8322.0 | 28.23% | 5392.6 | 18.54% | motif file (matrix) | svg |
| 15 | T C G A T A G C T G C A A C T G A C T G C G T A C G T A C T A G G A C T T A C G | ETS1(ETS)/Jurkat-ETS1-ChIP-Seq(GSE17954)/Homer | 1e-350 | -8.068e+02 | 0.0000 | 6237.0 | 21.16% | 3711.1 | 12.76% | motif file (matrix) | svg |
| 16 | C T G A T A G C T G A C T C A G C T A G G T C A C G T A T C A G A G C T T C A G | ETV4(ETS)/HepG2-ETV4-ChIP-Seq(ENCODE)/Homer | 1e-344 | -7.940e+02 | 0.0000 | 6633.0 | 22.50% | 4045.6 | 13.91% | motif file (matrix) | svg |
| 17 | T C G A C T G A T A G C T G A C T C A G T C A G C G T A C G T A T C A G A G C T | ETV1(ETS)/GIST48-ETV1-ChIP-Seq(GSE22441)/Homer | 1e-340 | -7.837e+02 | 0.0000 | 7183.0 | 24.37% | 4507.9 | 15.50% | motif file (matrix) | svg |
| 18 | G A T C C T G A A G T C C G A T C G A T G A T C A G T C A C T G A T C G A G C T | Elk4(ETS)/Hela-Elk4-ChIP-Seq(GSE31477)/Homer | 1e-304 | -7.012e+02 | 0.0000 | 3678.0 | 12.48% | 1888.7 | 6.49% | motif file (matrix) | svg |
| 19 | T G C A C T G A A G T C G T C A A C T G A C T G C G T A C G T A C T G A A G C T | EWS:FLI1-fusion(ETS)/SK\_N\_MC-EWS:FLI1-ChIP-Seq(SRA014231)/Homer | 1e-300 | -6.919e+02 | 0.0000 | 3590.0 | 12.18% | 1834.7 | 6.31% | motif file (matrix) | svg |
| 20 | T C G A T C G A T A G C G T A C T C A G T A C G C G T A C G T A T C A G A G C T | GABPA(ETS)/Jurkat-GABPa-ChIP-Seq(GSE17954)/Homer | 1e-290 | -6.696e+02 | 0.0000 | 5012.0 | 17.00% | 2926.1 | 10.06% | motif file (matrix) | svg |
| 21 | G A T C T C G A A G T C C G A T C G A T A G T C A T G C A C T G A T C G G A C T | Elk1(ETS)/Hela-Elk1-ChIP-Seq(GSE31477)/Homer | 1e-283 | -6.517e+02 | 0.0000 | 3737.0 | 12.68% | 1982.5 | 6.81% | motif file (matrix) | svg |
| 22 | T A G C A G T C T G A C A G T C C T A G A T C G A G T C C A T G T G A C A G T C G T A C A G T C A G T C G C A T C T A G A T C G G C A T A C T G A T C G G A T C | BORIS(Zf)/K562-CTCFL-ChIP-Seq(GSE32465)/Homer | 1e-281 | -6.470e+02 | 0.0000 | 2647.0 | 8.98% | 1218.5 | 4.19% | motif file (matrix) | svg |
| 23 | G A C T C G A T C T G A G T C A G A C T C G A T T C G A C G T A G C T A G C T A T G A C G T A C C G T A A C T G T G C A C G A T A C T G A C G T | Pitx1:Ebox(Homeobox,bHLH)/Hindlimb-Pitx1-ChIP-Seq(GSE41591)/Homer | 1e-249 | -5.755e+02 | 0.0000 | 1765.0 | 5.99% | 701.7 | 2.41% | motif file (matrix) | svg |
| 24 | T C G A A G C T A C G T A C G T A G T C A G T C A C G T A T C G G A C T A T C G | EWS:ERG-fusion(ETS)/CADO\_ES1-EWS:ERG-ChIP-Seq(SRA014231)/Homer | 1e-224 | -5.159e+02 | 0.0000 | 4055.0 | 13.76% | 2384.1 | 8.20% | motif file (matrix) | svg |
| 25 | C T G A A G T C C G A T A G C T A T G C G T A C A C G T A T C G C A G T G C A T | Elf4(ETS)/BMDM-Elf4-ChIP-Seq(GSE88699)/Homer | 1e-218 | -5.022e+02 | 0.0000 | 4987.0 | 16.92% | 3142.4 | 10.80% | motif file (matrix) | svg |
| 26 | T G C A T C G A T A G C G T A C T C A G C T A G G T C A G C T A T C A G G A C T | ETS(ETS)/Promoter/Homer | 1e-212 | -4.903e+02 | 0.0000 | 2082.0 | 7.06% | 969.5 | 3.33% | motif file (matrix) | svg |
| 27 | C T G A T G C A T A G C T G A C T A C G T C A G C T G A G C T A T C A G G A C T | ELF1(ETS)/Jurkat-ELF1-ChIP-Seq(SRA014231)/Homer | 1e-194 | -4.484e+02 | 0.0000 | 3025.0 | 10.26% | 1685.2 | 5.79% | motif file (matrix) | svg |
| 28 | C G T A T A G C T A G C T G C A A C T G C T A G C G T A C G T A T C A G G A C T | EHF(ETS)/LoVo-EHF-ChIP-Seq(GSE49402)/Homer | 1e-176 | -4.067e+02 | 0.0000 | 6252.0 | 21.21% | 4366.5 | 15.01% | motif file (matrix) | svg |
| 29 | T A C G T C G A C A G T A C T G G C T A A T G C C G A T G T A C C G T A A C T G T A G C C G T A | NF-E2(bZIP)/K562-NFE2-ChIP-Seq(GSE31477)/Homer | 1e-172 | -3.982e+02 | 0.0000 | 602.0 | 2.04% | 146.5 | 0.50% | motif file (matrix) | svg |
| 30 | T A C G T G C A A G T C C G T A A C G T T G A C A C G T A C T G A C T G G C A T | TCF4(bHLH)/SHSY5Y-TCF4-ChIP-Seq(GSE96915)/Homer | 1e-158 | -3.656e+02 | 0.0000 | 8283.0 | 28.10% | 6241.1 | 21.45% | motif file (matrix) | svg |
| 31 | G T C A G C A T A C T G G T A C G A C T A C T G G C T A A T C G C A G T G T A C C G T A A G C T | Nrf2(bZIP)/Lymphoblast-Nrf2-ChIP-Seq(GSE37589)/Homer | 1e-151 | -3.497e+02 | 0.0000 | 537.0 | 1.82% | 132.4 | 0.46% | motif file (matrix) | svg |
| 32 | C T A G T C G A T G A C A G T C C G T A A C T G G T A C A C G T A C T G A C T G | BHLHA15(bHLH)/NIH3T3-BHLHB8.HA-ChIP-Seq(GSE119782)/Homer | 1e-141 | -3.263e+02 | 0.0000 | 7966.0 | 27.02% | 6057.9 | 20.82% | motif file (matrix) | svg |
| 33 | C G T A C G T A C G T A G C A T G C A T T A C G G T A C G A C T A C T G C G T A A T C G A C G T G T A C C G T A A G C T | Bach1(bZIP)/K562-Bach1-ChIP-Seq(GSE31477)/Homer | 1e-139 | -3.216e+02 | 0.0000 | 595.0 | 2.02% | 170.4 | 0.59% | motif file (matrix) | svg |
| 34 | T G A C A G T C C G T A A C T G G T A C A C G T A C T G A C G T G A C T G A T C | Twist2(bHLH)/Myoblast-Twist2.Ty1-ChIP-Seq(GSE127998)/Homer | 1e-138 | -3.188e+02 | 0.0000 | 9618.0 | 32.63% | 7581.0 | 26.06% | motif file (matrix) | svg |
| 35 | T A G C C G T A C T G A T A C G C G T A A C G T A C T G A C T G A G T C T A C G C T A G G T A C | YY1(Zf)/Promoter/Homer | 1e-138 | -3.180e+02 | 0.0000 | 1024.0 | 3.47% | 415.8 | 1.43% | motif file (matrix) | svg |
| 36 | A T G C T C A G T C G A G C A T A C T G C G T A A G T C T C A G G A C T T G A C C G T A A G C T | Atf2(bZIP)/3T3L1-Atf2-ChIP-Seq(GSE56872)/Homer | 1e-136 | -3.144e+02 | 0.0000 | 1883.0 | 6.39% | 1000.4 | 3.44% | motif file (matrix) | svg |
| 37 | T A G C C T A G T C G A G A C T A C T G C T G A A G T C T C A G G C A T T G A C C T G A A G C T | Atf7(bZIP)/3T3L1-Atf7-ChIP-Seq(GSE56872)/Homer | 1e-135 | -3.111e+02 | 0.0000 | 2486.0 | 8.43% | 1453.6 | 5.00% | motif file (matrix) | svg |
| 38 | T G C A C T G A A T G C G T C A A C T G A C T G C G T A C G T A C T A G A G C T | Ets1-distal(ETS)/CD4+-PolII-ChIP-Seq(Barski\_et\_al.)/Homer | 1e-132 | -3.048e+02 | 0.0000 | 1612.0 | 5.47% | 817.0 | 2.81% | motif file (matrix) | svg |
| 39 | A G C T C T A G T G A C C G T A A C G T C G A T A G T C A G T C C T G A C A T G | TEAD3(TEA)/HepG2-TEAD3-ChIP-Seq(Encode)/Homer | 1e-127 | -2.936e+02 | 0.0000 | 5019.0 | 17.03% | 3550.7 | 12.20% | motif file (matrix) | svg |
| 40 | T C G A G C A T A C T G C T G A A G T C T C A G G A C T G T A C C G T A A G C T A G T C G A T C | c-Jun-CRE(bZIP)/K562-cJun-ChIP-Seq(GSE31477)/Homer | 1e-126 | -2.912e+02 | 0.0000 | 1749.0 | 5.93% | 929.0 | 3.19% | motif file (matrix) | svg |
| 41 | T G A C A G T C C T G A T G A C C G T A A C G T A C G T A G T C A G T C C G T A | TEAD1(TEAD)/HepG2-TEAD1-ChIP-Seq(Encode)/Homer | 1e-123 | -2.848e+02 | 0.0000 | 4317.0 | 14.64% | 2974.7 | 10.23% | motif file (matrix) | svg |
| 42 | T G C A T C G A T C G A A C G T C A T G C G T A A G T C T C A G A C G T G T A C C G T A A G C T | CREB5(bZIP)/LNCaP-CREB5.V5-ChIP-Seq(GSE137775)/Homer | 1e-122 | -2.815e+02 | 0.0000 | 2131.0 | 7.23% | 1223.1 | 4.20% | motif file (matrix) | svg |
| 43 | T C G A T G A C G T A C C G T A C A G T T G A C A C G T A C T G A G C T A G C T | NeuroG2(bHLH)/Fibroblast-NeuroG2-ChIP-Seq(GSE75910)/Homer | 1e-122 | -2.813e+02 | 0.0000 | 8244.0 | 27.97% | 6432.4 | 22.11% | motif file (matrix) | svg |
| 44 | G A T C G T A C C G A T A C T G A C T G C G T A C G T A A C G T A C T G G A T C | TEAD(TEA)/Fibroblast-PU.1-ChIP-Seq(Unpublished)/Homer | 1e-118 | -2.732e+02 | 0.0000 | 3138.0 | 10.64% | 2024.3 | 6.96% | motif file (matrix) | svg |
| 45 | T C A G A C G T T C G A T A G C A G T C C G T A A C T G G T A C A C G T A C T G A T C G A G T C | Atoh1(bHLH)/Cerebellum-Atoh1-ChIP-Seq(GSE22111)/Homer | 1e-116 | -2.683e+02 | 0.0000 | 6298.0 | 21.36% | 4723.3 | 16.24% | motif file (matrix) | svg |
| 46 | T A G C T A G C G C A T C A T G A C T G G C T A C G T A A C G T A C T G G A T C | TEAD4(TEA)/Tropoblast-Tead4-ChIP-Seq(GSE37350)/Homer | 1e-115 | -2.665e+02 | 0.0000 | 3735.0 | 12.67% | 2525.4 | 8.68% | motif file (matrix) | svg |
| 47 | T C A G T A G C G A C T C A T G C T G A A T C G G C A T G T A C C G T A A C T G T A G C T G C A | MafK(bZIP)/C2C12-MafK-ChIP-Seq(GSE36030)/Homer | 1e-115 | -2.656e+02 | 0.0000 | 1317.0 | 4.47% | 650.4 | 2.24% | motif file (matrix) | svg |
| 48 | C G T A C G T A C G T A G C A T G C A T A C T G G T A C G A C T C T A G G C T A T A C G G A C T T G A C C G T A A G C T | NFE2L2(bZIP)/HepG2-NFE2L2-ChIP-Seq(Encode)/Homer | 1e-114 | -2.631e+02 | 0.0000 | 516.0 | 1.75% | 154.3 | 0.53% | motif file (matrix) | svg |
| 49 | C T G A A T G C C G T A A C G T A G T C A G T C A C G T A C T G A T C G G C A T | SPDEF(ETS)/VCaP-SPDEF-ChIP-Seq(SRA014231)/Homer | 1e-112 | -2.602e+02 | 0.0000 | 4658.0 | 15.80% | 3316.9 | 11.40% | motif file (matrix) | svg |
| 50 | C G T A T G A C T A G C T G C A A C T G A C T G C G T A C G T A T C A G G A C T | ELF3(ETS)/PDAC-ELF3-ChIP-Seq(GSE64557)/Homer | 1e-112 | -2.596e+02 | 0.0000 | 3467.0 | 11.76% | 2317.6 | 7.97% | motif file (matrix) | svg |
| 51 | T C A G T G A C G T A C C G T A A C G T T G A C A C G T T C A G A G C T G A C T | NeuroD1(bHLH)/Islet-NeuroD1-ChIP-Seq(GSE30298)/Homer | 1e-111 | -2.577e+02 | 0.0000 | 4457.0 | 15.12% | 3150.9 | 10.83% | motif file (matrix) | svg |
| 52 | T A C G T C G A G A C T A C T G C T G A A G T C T C A G G A C T T G A C C T G A | Atf1(bZIP)/K562-ATF1-ChIP-Seq(GSE31477)/Homer | 1e-102 | -2.371e+02 | 0.0000 | 3292.0 | 11.17% | 2216.6 | 7.62% | motif file (matrix) | svg |
| 53 | C T G A T G A C T G A C C G T A A C G T T G A C A G C T C T A G A C G T G A C T | Olig2(bHLH)/Neuron-Olig2-ChIP-Seq(GSE30882)/Homer | 1e-93 | -2.145e+02 | 0.0000 | 9301.0 | 31.55% | 7619.5 | 26.19% | motif file (matrix) | svg |
| 54 | T C G A A C G T A C T G C T G A A G T C T C A G A G C T G T A C C G T A A G C T G A T C T C G A | JunD(bZIP)/K562-JunD-ChIP-Seq/Homer | 1e-91 | -2.109e+02 | 0.0000 | 748.0 | 2.54% | 318.3 | 1.09% | motif file (matrix) | svg |
| 55 | G C T A A G T C T A C G T G C A A T C G T C A G G C T A T C G A T C A G A G C T | ELF5(ETS)/T47D-ELF5-ChIP-Seq(GSE30407)/Homer | 1e-91 | -2.099e+02 | 0.0000 | 3498.0 | 11.87% | 2444.8 | 8.40% | motif file (matrix) | svg |
| 56 | A T G C G A T C C G A T C T A G A C T G G C T A C G T A A G C T A C T G A G C T | TEAD2(TEA)/Py2T-Tead2-ChIP-Seq(GSE55709)/Homer | 1e-86 | -1.983e+02 | 0.0000 | 2323.0 | 7.88% | 1499.2 | 5.15% | motif file (matrix) | svg |
| 57 | C G A T T G C A T G C A G A T C C G T A A C T G T G A C G A C T C A T G A C T G | Tcf21(bHLH)/ArterySmoothMuscle-Tcf21-ChIP-Seq(GSE61369)/Homer | 1e-79 | -1.836e+02 | 0.0000 | 5991.0 | 20.32% | 4692.3 | 16.13% | motif file (matrix) | svg |
| 58 | A T G C A T C G T A C G A G C T A T C G C T G A A G T C C T A G A G C T A T G C C T G A A T G C | CRE(bZIP)/Promoter/Homer | 1e-79 | -1.822e+02 | 0.0000 | 1592.0 | 5.40% | 950.4 | 3.27% | motif file (matrix) | svg |
| 59 | G T C A T G C A G C T A A G T C C G T A A C T G T G A C G C A T T C A G C A G T | Ap4(bHLH)/AML-Tfap4-ChIP-Seq(GSE45738)/Homer | 1e-66 | -1.533e+02 | 0.0000 | 7264.0 | 24.64% | 5958.2 | 20.48% | motif file (matrix) | svg |
| 60 | T G A C G T A C C G T A A C T G T G A C C G A T A C T G A T C G A G C T T A C G T C G A T A G C G T A C C G T A A T C G T G A C G C A T A C T G A C T G A T G C | Twist(bHLH)/HMLE-TWIST1-ChIP-Seq(Chang\_et\_al)/Homer | 1e-65 | -1.508e+02 | 0.0000 | 984.0 | 3.34% | 536.7 | 1.84% | motif file (matrix) | svg |
| 61 | C T G A T C A G G T A C G C T A A C T G T G A C G C A T C A T G | SCL(bHLH)/HPC7-Scl-ChIP-Seq(GSE13511)/Homer | 1e-62 | -1.449e+02 | 0.0000 | 20374.0 | 69.11% | 18758.5 | 64.48% | motif file (matrix) | svg |
| 62 | C G T A T A C G T C G A A C T G A C T G C G T A C G T A T A C G A G C T T A C G | PU.1(ETS)/ThioMac-PU.1-ChIP-Seq(GSE21512)/Homer | 1e-60 | -1.386e+02 | 0.0000 | 2127.0 | 7.22% | 1455.0 | 5.00% | motif file (matrix) | svg |
| 63 | T C G A A C G T A C G T C T G A G A T C T C A G G A C T G T C A C G T A A G C T G T C A C T A G A G C T A C G T T C G A | NFIL3(bZIP)/HepG2-NFIL3-ChIP-Seq(Encode)/Homer | 1e-59 | -1.369e+02 | 0.0000 | 3218.0 | 10.92% | 2382.2 | 8.19% | motif file (matrix) | svg |
| 64 | T A C G T A C G G T A C A T C G T A C G T A C G G T C A C T G A C G T A G A C T | E2F4(E2F)/K562-E2F4-ChIP-Seq(GSE31477)/Homer | 1e-58 | -1.351e+02 | 0.0000 | 3268.0 | 11.09% | 2430.5 | 8.35% | motif file (matrix) | svg |
| 65 | T G C A C T G A A T G C G T C A A C G T A T G C A C G T A C T G A C T G T G C A | ZBTB18(Zf)/HEK293-ZBTB18.GFP-ChIP-Seq(GSE58341)/Homer | 1e-56 | -1.303e+02 | 0.0000 | 3012.0 | 10.22% | 2222.4 | 7.64% | motif file (matrix) | svg |
| 66 | T C A G A G C T A T G C C G T A A G C T T C A G C A G T A C T G C T G A A G T C | MITF(bHLH)/MastCells-MITF-ChIP-Seq(GSE48085)/Homer | 1e-55 | -1.283e+02 | 0.0000 | 3633.0 | 12.32% | 2768.8 | 9.52% | motif file (matrix) | svg |
| 67 | C T A G C A G T T G A C C G T A G A T C T C A G G A C T C A T G | BMAL1(bHLH)/Liver-Bmal1-ChIP-Seq(GSE39860)/Homer | 1e-47 | -1.098e+02 | 0.0000 | 7354.0 | 24.95% | 6225.5 | 21.40% | motif file (matrix) | svg |
| 68 | C G A T T G C A G T A C C G T A A G T C C T A G G A C T C A T G | NPAS(bHLH)/Liver-NPAS-ChIP-Seq(GSE39860)/Homer | 1e-45 | -1.053e+02 | 0.0000 | 6864.0 | 23.28% | 5789.1 | 19.90% | motif file (matrix) | svg |
| 69 | C T G A T C A G G T A C T G C A A G T C C G T A A G T C A C T G A C G T A C T G | MNT(bHLH)/HepG2-MNT-ChIP-Seq(Encode)/Homer | 1e-43 | -1.007e+02 | 0.0000 | 4232.0 | 14.36% | 3394.7 | 11.67% | motif file (matrix) | svg |
| 70 | T C G A T G A C A G T C C G T A A G T C C T A G A C G T A C T G A C T G A G C T A G T C G C A T | Max(bHLH)/K562-Max-ChIP-Seq(GSE31477)/Homer | 1e-42 | -9.761e+01 | 0.0000 | 3042.0 | 10.32% | 2344.2 | 8.06% | motif file (matrix) | svg |
| 71 | T C G A T A G C G T C A A C T G C T A G C G T A C G A T A C T G A C G T A C T G A C T G A C G T | ETS:RUNX(ETS,Runt)/Jurkat-RUNX1-ChIP-Seq(GSE17954)/Homer | 1e-41 | -9.567e+01 | 0.0000 | 487.0 | 1.65% | 243.8 | 0.84% | motif file (matrix) | svg |
| 72 | G A C T C T A G G A T C C A G T A C T G C T G A A T G C G C A T A T G C C T G A | MafA(bZIP)/Islet-MafA-ChIP-Seq(GSE30298)/Homer | 1e-39 | -9.047e+01 | 0.0000 | 4220.0 | 14.32% | 3423.4 | 11.77% | motif file (matrix) | svg |
| 73 | T A G C C G A T A C G T A G C T A G C T A G T C A T G C A G T C A C T G A T G C A T G C G C T A | E2F7(E2F)/Hela-E2F7-ChIP-Seq(GSE32673)/Homer | 1e-37 | -8.672e+01 | 0.0000 | 890.0 | 3.02% | 554.1 | 1.90% | motif file (matrix) | svg |
| 74 | C T A G A G T C T A C G T A C G T G A C C G T A A C T G T A G C G C A T C A T G A T G C A G C T | Ascl1(bHLH)/NeuralTubes-Ascl1-ChIP-Seq(GSE55840)/Homer | 1e-37 | -8.632e+01 | 0.0000 | 7906.0 | 26.82% | 6862.4 | 23.59% | motif file (matrix) | svg |
| 75 | C G T A G A C T C G A T A T C G G T A C G C A T C A T G C G T A T A C G G C A T G T A C C G T A C A T G A T G C G C T A C T A G G C A T G C A T G C A T G A C T | MafB(bZIP)/BMM-Mafb-ChIP-Seq(GSE75722)/Homer | 1e-35 | -8.224e+01 | 0.0000 | 2087.0 | 7.08% | 1558.6 | 5.36% | motif file (matrix) | svg |
| 76 | T C A G A C G T A G T C T C G A A G T C T C A G G C A T C T A G C T A G A G C T | Usf2(bHLH)/C2C12-Usf2-ChIP-Seq(GSE36030)/Homer | 1e-35 | -8.206e+01 | 0.0000 | 1451.0 | 4.92% | 1018.7 | 3.50% | motif file (matrix) | svg |
| 77 | T C A G C G T A A G T C A G C T C G T A A G T C C T G A C G T A A G T C G C A T A G T C A G T C A G T C C T G A A C T G T G C A T C G A C A T G A T C G G A T C | Ronin(THAP)/ES-Thap11-ChIP-Seq(GSE51522)/Homer | 1e-35 | -8.196e+01 | 0.0000 | 279.0 | 0.95% | 117.0 | 0.40% | motif file (matrix) | svg |
| 78 | T A G C G C T A T C G A C T G A A G T C A G T C C T G A A G T C C G T A C T A G | RUNX(Runt)/HPC7-Runx1-ChIP-Seq(GSE22178)/Homer | 1e-34 | -7.914e+01 | 0.0000 | 2774.0 | 9.41% | 2170.0 | 7.46% | motif file (matrix) | svg |
| 79 | T C G A G C A T A C G T C T A G G T A C T C G A G C A T T G A C T C G A A C G T | Chop(bZIP)/MEF-Chop-ChIP-Seq(GSE35681)/Homer | 1e-34 | -7.906e+01 | 0.0000 | 1314.0 | 4.46% | 911.5 | 3.13% | motif file (matrix) | svg |
| 80 | T C A G A G C T A T G C C G T A A G T C T C A G A C G T A T C G T C G A A G T C G A T C T G A C | TFE3(bHLH)/MEF-TFE3-ChIP-Seq(GSE75757)/Homer | 1e-31 | -7.190e+01 | 0.0000 | 434.0 | 1.47% | 231.2 | 0.79% | motif file (matrix) | svg |
| 81 | T G C A T A G C G A C T T G C A T G A C T G C A C G T A A G C T A G C T A G T C A G T C G T A C | GFY(?)/Promoter/Homer | 1e-30 | -7.002e+01 | 0.0000 | 376.0 | 1.28% | 192.6 | 0.66% | motif file (matrix) | svg |
| 82 | C T A G G C A T G A T C C G T A A G T C T C A G G A C T C T A G | CLOCK(bHLH)/Liver-Clock-ChIP-Seq(GSE39860)/Homer | 1e-29 | -6.900e+01 | 0.0000 | 2605.0 | 8.84% | 2055.7 | 7.07% | motif file (matrix) | svg |
| 83 | G C T A C T G A T C G A A G T C A G T C C T G A A G T C G T C A C T G A T G C A | RUNX1(Runt)/Jurkat-RUNX1-ChIP-Seq(GSE29180)/Homer | 1e-29 | -6.780e+01 | 0.0000 | 3995.0 | 13.55% | 3316.0 | 11.40% | motif file (matrix) | svg |
| 84 | A G T C G A C T C A G T G T A C A G T C A T C G T C A G A C T G G T C A C G T A | Stat3(Stat)/mES-Stat3-ChIP-Seq(GSE11431)/Homer | 1e-28 | -6.608e+01 | 0.0000 | 1981.0 | 6.72% | 1515.0 | 5.21% | motif file (matrix) | svg |
| 85 | C G A T G A C T C G A T T C A G G A C T A C G T C A G T C T G A G A C T G A C T A G C T C G A T A C T G A T C G G T A C G C T A | NF1:FOXA1(CTF,Forkhead)/LNCAP-FOXA1-ChIP-Seq(GSE27824)/Homer | 1e-28 | -6.573e+01 | 0.0000 | 351.0 | 1.19% | 179.1 | 0.62% | motif file (matrix) | svg |
| 86 | T A C G T A C G G T A C A T C G A C T G T A C G T C G A C T G A T C G A A T C G | E2F6(E2F)/Hela-E2F6-ChIP-Seq(GSE31477)/Homer | 1e-28 | -6.558e+01 | 0.0000 | 4143.0 | 14.05% | 3461.2 | 11.90% | motif file (matrix) | svg |
| 87 | T A C G T C G A T A G C A G T C C G T A A G T C C T A G G C A T A C T G A T C G | n-Myc(bHLH)/mES-nMyc-ChIP-Seq(GSE11431)/Homer | 1e-28 | -6.535e+01 | 0.0000 | 3485.0 | 11.82% | 2861.6 | 9.84% | motif file (matrix) | svg |
| 88 | T C G A C G T A A G T C A G C T C G T A A G T C T C G A G C T A G A C T C G A T A G T C A G T C A G T C C T G A T C A G T G C A T C G A C A G T A T C G A G T C | GFY-Staf(?,Zf)/Promoter/Homer | 1e-27 | -6.335e+01 | 0.0000 | 346.0 | 1.17% | 178.5 | 0.61% | motif file (matrix) | svg |
| 89 | T C G A A G T C C G T A A T C G A T G C C G A T A C T G A G T C A G C T A C T G | Tcf12(bHLH)/GM12878-Tcf12-ChIP-Seq(GSE32465)/Homer | 1e-27 | -6.249e+01 | 0.0000 | 5813.0 | 19.72% | 5027.5 | 17.28% | motif file (matrix) | svg |
| 90 | A C T G G A T C G A C T A C T G A C G T C A T G A C T G A C G T A G C T C G A T | RUNX-AML(Runt)/CD4+-PolII-ChIP-Seq(Barski\_et\_al.)/Homer | 1e-26 | -6.174e+01 | 0.0000 | 3012.0 | 10.22% | 2448.5 | 8.42% | motif file (matrix) | svg |
| 91 | A C G T T C G A T C G A A G T C G T C A T A C G A T G C A C G T A C T G A G C T | Myf5(bHLH)/GM-Myf5-ChIP-Seq(GSE24852)/Homer | 1e-26 | -6.134e+01 | 0.0000 | 4175.0 | 14.16% | 3512.0 | 12.07% | motif file (matrix) | svg |
| 92 | A T G C A T G C A T C G T A C G A G C T A G T C G C T A A G T C T C A G G A C T A C T G T C G A | E-box(bHLH)/Promoter/Homer | 1e-26 | -6.022e+01 | 0.0000 | 536.0 | 1.82% | 322.3 | 1.11% | motif file (matrix) | svg |
| 93 | T C G A A G T C C G T A A T C G T A G C A C G T A C T G A G C T A C G T A G T C | Ptf1a(bHLH)/Panc1-Ptf1a-ChIP-Seq(GSE47459)/Homer | 1e-25 | -5.972e+01 | 0.0000 | 12355.0 | 41.91% | 11311.0 | 38.88% | motif file (matrix) | svg |
| 94 | A C T G A C G T C A T G A T C G A T C G T G A C A C T G A T C G A T C G T G C A C T G A C G T A | E2F3(E2F)/MEF-E2F3-ChIP-Seq(GSE71376)/Homer | 1e-25 | -5.968e+01 | 0.0000 | 4400.0 | 14.93% | 3727.7 | 12.81% | motif file (matrix) | svg |
| 95 | T A C G C T G A T C G A C G A T C T A G C T A G T C G A C T G A T C G A T C G A C G T A T C G A G C A T C A T G C G T A T A C G G C A T T G A C C G T A A G C T | NFAT:AP1(RHD,bZIP)/Jurkat-NFATC1-ChIP-Seq(Jolma\_et\_al.)/Homer | 1e-25 | -5.896e+01 | 0.0000 | 829.0 | 2.81% | 556.4 | 1.91% | motif file (matrix) | svg |
| 96 | T G A C C T A G T C A G G T C A C G T A T C A G C G A T T C A G T C G A T G C A C T G A T A G C | PU.1-IRF(ETS:IRF)/Bcell-PU.1-ChIP-Seq(GSE21512)/Homer | 1e-25 | -5.776e+01 | 0.0000 | 5583.0 | 18.94% | 4839.6 | 16.64% | motif file (matrix) | svg |
| 97 | T G C A A G C T C A T G C G T A A G C T A C T G G A T C G T C A C G T A A G C T | Atf4(bZIP)/MEF-Atf4-ChIP-Seq(GSE35681)/Homer | 1e-25 | -5.767e+01 | 0.0000 | 1675.0 | 5.68% | 1275.3 | 4.38% | motif file (matrix) | svg |
| 98 | C G T A C T G A C G T A C T A G T C G A C T A G A C T G C G T A C G T A T A C G A G C T A T C G | SpiB(ETS)/OCILY3-SPIB-ChIP-Seq(GSE56857)/Homer | 1e-24 | -5.669e+01 | 0.0000 | 1146.0 | 3.89% | 824.1 | 2.83% | motif file (matrix) | svg |
| 99 | T A G C C G A T T A C G A C T G A G T C A C T G A T C G A T C G C G T A C T G A | E2F1(E2F)/Hela-E2F1-ChIP-Seq(GSE22478)/Homer | 1e-24 | -5.584e+01 | 0.0000 | 1993.0 | 6.76% | 1560.3 | 5.36% | motif file (matrix) | svg |
| 100 | C T A G T A C G G A T C G T A C C T G A A G C T T G C A G C T A C G T A G C A T G A T C G C T A | Hoxc9(Homeobox)/Ainv15-Hoxc9-ChIP-Seq(GSE21812)/Homer | 1e-23 | -5.411e+01 | 0.0000 | 2350.0 | 7.97% | 1884.2 | 6.48% | motif file (matrix) | svg |
| 101 | A G T C A C G T A C T G A G C T A C G T A C G T G T C A A G T C | Foxo1(Forkhead)/RAW-Foxo1-ChIP-Seq(Fan\_et\_al.)/Homer | 1e-23 | -5.368e+01 | 0.0000 | 7516.0 | 25.50% | 6692.4 | 23.00% | motif file (matrix) | svg |
| 102 | T A C G T C A G A G C T A T G C C G T A A G T C T C A G A C G T A C T G T C G A | USF1(bHLH)/GM12878-Usf1-ChIP-Seq(GSE32465)/Homer | 1e-22 | -5.269e+01 | 0.0000 | 2214.0 | 7.51% | 1768.7 | 6.08% | motif file (matrix) | svg |
| 103 | T G A C G C T A T C G A T G C A A G T C A G T C C G T A A G T C C G T A C T G A G C T A G T A C | RUNX2(Runt)/PCa-RUNX2-ChIP-Seq(GSE33889)/Homer | 1e-22 | -5.176e+01 | 0.0000 | 3193.0 | 10.83% | 2657.5 | 9.13% | motif file (matrix) | svg |
| 104 | T C G A G A C T A G C T T G A C A G C T G T A C T C A G G A T C A T C G T G C A A C T G C T G A | GFX(?)/Promoter/Homer | 1e-22 | -5.100e+01 | 0.0000 | 135.0 | 0.46% | 50.6 | 0.17% | motif file (matrix) | svg |
| 105 | G C T A C G A T C A T G A T G C A G T C A G T C G A C T T A C G T C G A C T A G A C T G T A G C | AP-2alpha(AP2)/Hela-AP2alpha-ChIP-Seq(GSE31477)/Homer | 1e-21 | -5.018e+01 | 0.0000 | 4294.0 | 14.57% | 3680.4 | 12.65% | motif file (matrix) | svg |
| 106 | A T C G A G T C A G T C G A C T A T G C C T G A C T A G A C T G T A C G G T A C C T G A C G A T | AP-2gamma(AP2)/MCF7-TFAP2C-ChIP-Seq(GSE21234)/Homer | 1e-21 | -4.989e+01 | 0.0000 | 5536.0 | 18.78% | 4843.7 | 16.65% | motif file (matrix) | svg |
| 107 | T G C A A C T G T A C G A T G C A G T C G A C T T C G A A T C G | ZNF711(Zf)/SHSY5Y-ZNF711-ChIP-Seq(GSE20673)/Homer | 1e-21 | -4.921e+01 | 0.0000 | 6437.0 | 21.84% | 5698.7 | 19.59% | motif file (matrix) | svg |
| 108 | T C G A T C G A A G T C C G T A C T A G T A G C A C G T A C T G | MyoG(bHLH)/C2C12-MyoG-ChIP-Seq(GSE36024)/Homer | 1e-20 | -4.739e+01 | 0.0000 | 5747.0 | 19.50% | 5058.6 | 17.39% | motif file (matrix) | svg |
| 109 | C T G A T C A G A G T C C G T A A T C G A T G C C G A T A C T G A G T C G A C T A T C G A G T C | MyoD(bHLH)/Myotube-MyoD-ChIP-Seq(GSE21614)/Homer | 1e-20 | -4.667e+01 | 0.0000 | 5212.0 | 17.68% | 4559.5 | 15.67% | motif file (matrix) | svg |
| 110 | A T G C T C G A A G T C A G C T A C G T G T A C A G T C G C T A C T A G C A T G G T C A C T G A T C A G A G T C | Stat3+il21(Stat)/CD4-Stat3-ChIP-Seq(GSE19198)/Homer | 1e-20 | -4.639e+01 | 0.0000 | 2812.0 | 9.54% | 2335.0 | 8.03% | motif file (matrix) | svg |
| 111 | T G C A G C A T A G C T G C A T A G T C A G T C A G T C C T G A A C T G T C G A T C G A C A G T A T C G A G T C G A T C | ZNF143\|STAF(Zf)/CUTLL-ZNF143-ChIP-Seq(GSE29600)/Homer | 1e-19 | -4.471e+01 | 0.0000 | 1054.0 | 3.58% | 779.0 | 2.68% | motif file (matrix) | svg |
| 112 | A T G C C T G A G A C T A C G T A C G T G T A C G A T C C G A T C T A G C A T G C G T A C G T A C T G A G A C T | STAT1(Stat)/HelaS3-STAT1-ChIP-Seq(GSE12782)/Homer | 1e-19 | -4.451e+01 | 0.0000 | 1128.0 | 3.83% | 842.8 | 2.90% | motif file (matrix) | svg |
| 113 | T C A G C T A G C T A G A C T G A C T G G A T C A C T G A C T G C T A G C T A G A G T C G A T C | KLF1(Zf)/HUDEP2-KLF1-CutnRun(GSE136251)/Homer | 1e-18 | -4.352e+01 | 0.0000 | 4250.0 | 14.42% | 3678.4 | 12.64% | motif file (matrix) | svg |
| 114 | T C A G T C A G T A G C A G T C C T G A A G T C C T A G A C G T A C T G A T C G | c-Myc(bHLH)/mES-cMyc-ChIP-Seq(GSE11431)/Homer | 1e-18 | -4.314e+01 | 0.0000 | 2249.0 | 7.63% | 1839.8 | 6.32% | motif file (matrix) | svg |
| 115 | A T C G A G C T A C T G A G T C A C T G A G T C C G T A A C G T A C T G A G T C A C T G A G T C | NRF(NRF)/Promoter/Homer | 1e-18 | -4.306e+01 | 0.0000 | 1189.0 | 4.03% | 899.1 | 3.09% | motif file (matrix) | svg |
| 116 | C T G A T C A G C A G T C T A G A C T G C T A G G A T C A T C G A C T G C T G A T C A G G A T C | Sp5(Zf)/mES-Sp5.Flag-ChIP-Seq(GSE72989)/Homer | 1e-18 | -4.249e+01 | 0.0000 | 5611.0 | 19.03% | 4963.4 | 17.06% | motif file (matrix) | svg |
| 117 | A T G C A G C T T C A G T G A C T C A G A T G C T G C A A C G T A T C G G A T C A C T G A G T C | NRF1(NRF)/MCF7-NRF1-ChIP-Seq(Unpublished)/Homer | 1e-17 | -4.138e+01 | 0.0000 | 1176.0 | 3.99% | 893.7 | 3.07% | motif file (matrix) | svg |
| 118 | C G T A C T A G A C T G A C T G G A C T C T A G C A G T C T A G C A T G G A T C | KLF5(Zf)/LoVo-KLF5-ChIP-Seq(GSE49402)/Homer | 1e-17 | -4.104e+01 | 0.0000 | 5859.0 | 19.88% | 5208.7 | 17.90% | motif file (matrix) | svg |
| 119 | C A T G G T A C C G T A A G T C C T A G A C G T A C T G G T A C A G T C A G C T | bHLHE40(bHLH)/HepG2-BHLHE40-ChIP-Seq(GSE31477)/Homer | 1e-17 | -4.104e+01 | 0.0000 | 1702.0 | 5.77% | 1357.0 | 4.66% | motif file (matrix) | svg |
| 120 | A G T C A T C G C T A G A G C T G A C T C T A G A G T C A G T C G C T A C A G T T C A G T C A G G A T C C T G A T C G A G A T C | RFX(HTH)/K562-RFX3-ChIP-Seq(SRA012198)/Homer | 1e-17 | -4.093e+01 | 0.0000 | 511.0 | 1.73% | 335.3 | 1.15% | motif file (matrix) | svg |
| 121 | A G T C C T A G C T A G A G T C G A T C G T A C A G T C C T A G A G T C A G T C A G T C G T A C | Sp2(Zf)/HEK293-Sp2.eGFP-ChIP-Seq(Encode)/Homer | 1e-17 | -4.044e+01 | 0.0000 | 7707.0 | 26.14% | 6974.2 | 23.97% | motif file (matrix) | svg |
| 122 | A C T G T G A C G T A C C G T A A G T C T A C G A C G T A C T G G T C A A G T C | NPAS2(bHLH)/Liver-NPAS2-ChIP-Seq(GSE39860)/Homer | 1e-17 | -4.027e+01 | 0.0000 | 4424.0 | 15.01% | 3861.9 | 13.27% | motif file (matrix) | svg |
| 123 | A G T C G A C T C A G T A C T G C T A G T G A C G C T A A T G C G C A T A T C G C G A T A C T G G A T C G T A C G T C A C T G A | NF1(CTF)/LNCAP-NF1-ChIP-Seq(Unpublished)/Homer | 1e-17 | -4.020e+01 | 0.0000 | 1508.0 | 5.12% | 1188.8 | 4.09% | motif file (matrix) | svg |
| 124 | T C G A C T A G A G T C A G T C C G T A C G T A A C G T T A G C T C A G T A C G | NFY(CCAAT)/Promoter/Homer | 1e-17 | -3.926e+01 | 0.0000 | 2993.0 | 10.15% | 2537.1 | 8.72% | motif file (matrix) | svg |
| 125 | C T G A C G A T C T A G C G T A A G C T C G A T C A G T C T G A G A C T C T A G C T A G A T G C | PBX2(Homeobox)/K562-PBX2-ChIP-Seq(Encode)/Homer | 1e-16 | -3.901e+01 | 0.0000 | 3586.0 | 12.16% | 3087.3 | 10.61% | motif file (matrix) | svg |
| 126 | T C A G C T A G A T G C G A C T T G C A C G T A A G C T A C G T C T G A T A C G | En1(Homeobox)/SUM149-EN1-ChIP-Seq(GSE120957)/Homer | 1e-16 | -3.882e+01 | 0.0000 | 7571.0 | 25.68% | 6857.9 | 23.57% | motif file (matrix) | svg |
| 127 | C T G A A T G C G C T A C G A T A T G C C G T A C G T A C G T A C T A G T A C G | Tcf3(HMG)/mES-Tcf3-ChIP-Seq(GSE11724)/Homer | 1e-16 | -3.812e+01 | 0.0000 | 1149.0 | 3.90% | 880.5 | 3.03% | motif file (matrix) | svg |
| 128 | C T A G T C A G C A G T T C A G A C T G A C T G G A T C C T A G A C T G C T A G T C A G A T G C | KLF14(Zf)/HEK293-KLF14.GFP-ChIP-Seq(GSE58341)/Homer | 1e-15 | -3.556e+01 | 0.0000 | 8901.0 | 30.19% | 8165.6 | 28.07% | motif file (matrix) | svg |
| 129 | A G C T A C G T A C T G A T G C A G T C C G T A C T G A T A C G | NF1-halfsite(CTF)/LNCaP-NF1-ChIP-Seq(Unpublished)/Homer | 1e-14 | -3.335e+01 | 0.0000 | 7630.0 | 25.88% | 6961.4 | 23.93% | motif file (matrix) | svg |
| 130 | C G T A A T G C C G A T A C G T A G T C C G T A C G T A C G T A C T A G A T C G | TCFL2(HMG)/K562-TCF7L2-ChIP-Seq(GSE29196)/Homer | 1e-14 | -3.296e+01 | 0.0000 | 377.0 | 1.28% | 243.6 | 0.84% | motif file (matrix) | svg |
| 131 | C G T A C A T G C A T G A C T G C T A G T C G A G C A T C G A T A G C T A G T C G A T C G T A C | NFkB-p65(RHD)/GM12787-p65-ChIP-Seq(GSE19485)/Homer | 1e-13 | -3.196e+01 | 0.0000 | 2106.0 | 7.14% | 1762.2 | 6.06% | motif file (matrix) | svg |
| 132 | C G A T C T G A C G T A C A G T A G T C G A T C G A T C A C T G | Pitx1(Homeobox)/Chicken-Pitx1-ChIP-Seq(GSE38910)/Homer | 1e-13 | -3.129e+01 | 0.0000 | 14575.0 | 49.44% | 13746.8 | 47.25% | motif file (matrix) | svg |
| 133 | A C T G C A T G G C T A T C G A G C T A A G C T A G C T G T A C A G T C T G A C | NFkB-p65-Rel(RHD)/ThioMac-LPS-Expression(GSE23622)/Homer | 1e-13 | -3.073e+01 | 0.0000 | 353.0 | 1.20% | 228.3 | 0.78% | motif file (matrix) | svg |
| 134 | A C T G T C A G A G C T G A C T C A T G A G T C A G T C G C T A C G A T C T A G T C A G G T A C C T G A T C G A | Rfx1(HTH)/NPC-H3K4me1-ChIP-Seq(GSE16256)/Homer | 1e-13 | -3.003e+01 | 0.0000 | 906.0 | 3.07% | 695.4 | 2.39% | motif file (matrix) | svg |
| 135 | G A C T G T A C T G C A A C G T G A T C G C T A T C G A A C G T A G T C C G T A | Pdx1(Homeobox)/Islet-Pdx1-ChIP-Seq(SRA008281)/Homer | 1e-13 | -3.002e+01 | 0.0000 | 4173.0 | 14.16% | 3696.7 | 12.71% | motif file (matrix) | svg |
| 136 | C T A G A G C T G A C T C A T G A G T C A G T C G T C A C A G T C T A G T C A G G T A C C T G A T C G A G A T C T G A C | Rfx2(HTH)/LoVo-RFX2-ChIP-Seq(GSE49402)/Homer | 1e-12 | -2.933e+01 | 0.0000 | 521.0 | 1.77% | 368.4 | 1.27% | motif file (matrix) | svg |
| 137 | T A C G C T A G A T G C G A T C G T A C A G T C C T A G A G T C A G T C A G T C G T A C A G T C | Sp1(Zf)/Promoter/Homer | 1e-12 | -2.860e+01 | 0.0000 | 2682.0 | 9.10% | 2311.8 | 7.95% | motif file (matrix) | svg |
| 138 | A G T C G A C T A G C T C G A T A T C G G C T A C G A T A T C G C G A T A C T G T A C G A C G T | Tcf7(HMG)/GM12878-TCF7-ChIP-Seq(Encode)/Homer | 1e-12 | -2.849e+01 | 0.0000 | 1456.0 | 4.94% | 1189.1 | 4.09% | motif file (matrix) | svg |
| 139 | G A C T C T A G C T A G C T A G A C T G T C G A C T G A C T A G C T A G C T A G G T A C G T C A | ZNF467(Zf)/HEK293-ZNF467.GFP-ChIP-Seq(GSE58341)/Homer | 1e-12 | -2.803e+01 | 0.0000 | 4730.0 | 16.05% | 4238.3 | 14.57% | motif file (matrix) | svg |
| 140 | A T G C A G T C G T A C A G C T T C G A C T A G G A T C C T G A G T C A A G T C G C T A T C A G | Rfx5(HTH)/GM12878-Rfx5-ChIP-Seq(GSE31477)/Homer | 1e-11 | -2.757e+01 | 0.0000 | 1195.0 | 4.05% | 959.5 | 3.30% | motif file (matrix) | svg |
| 141 | T A C G T C A G G A T C G T A C T C G A G A C T G C T A G C T A G C T A C G T A G A T C G T C A | CDX4(Homeobox)/ZebrafishEmbryos-Cdx4.Myc-ChIP-Seq(GSE48254)/Homer | 1e-11 | -2.700e+01 | 0.0000 | 3873.0 | 13.14% | 3436.9 | 11.81% | motif file (matrix) | svg |
| 142 | T C A G T G A C G T A C T G C A G T A C C T A G G T A C A T G C A G T C G T C A A G T C G A C T | Klf9(Zf)/GBM-Klf9-ChIP-Seq(GSE62211)/Homer | 1e-11 | -2.689e+01 | 0.0000 | 1596.0 | 5.41% | 1323.6 | 4.55% | motif file (matrix) | svg |
| 143 | A G T C G C A T C G T A C G T A G T A C A C G T A C T G G A T C G A T C T C G A | BMYB(HTH)/Hela-BMYB-ChIP-Seq(GSE27030)/Homer | 1e-11 | -2.662e+01 | 0.0000 | 7081.0 | 24.02% | 6497.3 | 22.33% | motif file (matrix) | svg |
| 144 | T A C G T C A G T G C A A G C T T G A C A G C T A G T C A C T G G A T C A C T G T C G A A C T G C T G A C T G A A T G C | ZBTB33(Zf)/GM12878-ZBTB33-ChIP-Seq(GSE32465)/Homer | 1e-11 | -2.628e+01 | 0.0000 | 310.0 | 1.05% | 202.2 | 0.70% | motif file (matrix) | svg |
| 145 | T C A G T A C G G A T C G T A C T C G A C G A T C T G A C G T A G C T A C G T A | Hoxd11(Homeobox)/ChickenMSG-Hoxd11.Flag-ChIP-Seq(GSE86088)/Homer | 1e-11 | -2.604e+01 | 0.0000 | 9718.0 | 32.97% | 9053.8 | 31.12% | motif file (matrix) | svg |
| 146 | T C G A T A G C T G A C C T G A A G T C A C T G G A C T C A T G | c-Myc(bHLH)/LNCAP-cMyc-ChIP-Seq(Unpublished)/Homer | 1e-11 | -2.577e+01 | 0.0000 | 3069.0 | 10.41% | 2690.3 | 9.25% | motif file (matrix) | svg |
| 147 | A G C T A G C T T A G C A T C G A G T C A C T G A T G C A T C G T C G A C T G A T C G A C T G A | E2F(E2F)/Hela-CellCycle-Expression/Homer | 1e-11 | -2.574e+01 | 0.0000 | 262.0 | 0.89% | 165.5 | 0.57% | motif file (matrix) | svg |
| 148 | T A C G T A G C G C T A C G A T C T A G A C G T C A G T C A G T G C T A A G T C G T C A G C A T | FOXK2(Forkhead)/U2OS-FOXK2-ChIP-Seq(E-MTAB-2204)/Homer | 1e-10 | -2.443e+01 | 0.0000 | 2519.0 | 8.55% | 2186.9 | 7.52% | motif file (matrix) | svg |
| 149 | A T G C A G T C A G C T A G C T A C G T A T C G C G T A C G A T T A G C G A C T | LEF1(HMG)/H1-LEF1-ChIP-Seq(GSE64758)/Homer | 1e-10 | -2.413e+01 | 0.0000 | 2829.0 | 9.60% | 2477.2 | 8.52% | motif file (matrix) | svg |
| 150 | T C A G G A C T C A G T C T G A A G C T C T A G G A C T T G C A C T G A A G T C | HLF(bZIP)/HSC-HLF.Flag-ChIP-Seq(GSE69817)/Homer | 1e-10 | -2.364e+01 | 0.0000 | 3577.0 | 12.13% | 3183.2 | 10.94% | motif file (matrix) | svg |
| 151 | A G T C C G A T A C T G A T C G T G A C G C T A C A T G A T C G T G A C C G A T A C T G T A G C G T A C G T C A | Tlx?(NR)/NPC-H3K4me1-ChIP-Seq(GSE16256)/Homer | 1e-10 | -2.325e+01 | 0.0000 | 1521.0 | 5.16% | 1273.6 | 4.38% | motif file (matrix) | svg |
| 152 | G A C T C A G T A G C T C G A T A G T C G A T C A G T C C G T A A T G C T C A G | Rbpj1(?)/Panc1-Rbpj1-ChIP-Seq(GSE47459)/Homer | 1e-9 | -2.253e+01 | 0.0000 | 5177.0 | 17.56% | 4712.4 | 16.20% | motif file (matrix) | svg |
| 153 | G T A C C A G T A C T G A C T G A C T G G A T C A C T G A C G T A C T G A C T G A G T C G A T C | KLF6(Zf)/PDAC-KLF6-ChIP-Seq(GSE64557)/Homer | 1e-9 | -2.252e+01 | 0.0000 | 5498.0 | 18.65% | 5019.4 | 17.25% | motif file (matrix) | svg |
| 154 | G A C T T C A G C T A G A G T C A G T C G T A C A G T C C T G A A G T C A G T C A G T C G A C T A G T C A C T G A T G C | KLF3(Zf)/MEF-Klf3-ChIP-Seq(GSE44748)/Homer | 1e-9 | -2.210e+01 | 0.0000 | 2367.0 | 8.03% | 2060.3 | 7.08% | motif file (matrix) | svg |
| 155 | A T G C A G T C G C T A C G A T C G T A G C A T G C T A C G A T C T A G C A T G T G A C G T C A | CArG(MADS)/PUER-Srf-ChIP-Seq(Sullivan\_et\_al.)/Homer | 1e-9 | -2.194e+01 | 0.0000 | 993.0 | 3.37% | 802.9 | 2.76% | motif file (matrix) | svg |
| 156 | G C T A T C G A C G T A C T A G A G C T G T C A G T C A C G T A A G T C C G T A | FOXA1(Forkhead)/LNCAP-FOXA1-ChIP-Seq(GSE27824)/Homer | 1e-9 | -2.184e+01 | 0.0000 | 4924.0 | 16.70% | 4477.3 | 15.39% | motif file (matrix) | svg |
| 157 | T A C G A T C G G A T C G T C A C T G A G C A T C G A T G C T A T C G A G C T A | Unknown(Homeobox)/Limb-p300-ChIP-Seq/Homer | 1e-9 | -2.160e+01 | 0.0000 | 2832.0 | 9.61% | 2498.7 | 8.59% | motif file (matrix) | svg |
| 158 | A C G T C T A G A G C T A C G T A C G T C T G A A G T C G A C T A G C T C G T A | FOXM1(Forkhead)/MCF7-FOXM1-ChIP-Seq(GSE72977)/Homer | 1e-9 | -2.117e+01 | 0.0000 | 4195.0 | 14.23% | 3789.3 | 13.02% | motif file (matrix) | svg |
| 159 | C T G A C A G T C T G A A G T C C T A G G A C T A T C G G T A C | HIF-1b(HLH)/T47D-HIF1b-ChIP-Seq(GSE59937)/Homer | 1e-9 | -2.096e+01 | 0.0000 | 6447.0 | 21.87% | 5946.2 | 20.44% | motif file (matrix) | svg |
| 160 | C T A G A C T G T G C A G T C A A T G C C G T A A T C G A T G C A G T C C T A G | ZNF341(Zf)/EBV-ZNF341-ChIP-Seq(GSE113194)/Homer | 1e-9 | -2.095e+01 | 0.0000 | 4074.0 | 13.82% | 3676.4 | 12.64% | motif file (matrix) | svg |
| 161 | C A T G C T A G A G C T G A C T C A T G A G T C G A T C G C T A C G A T C T A G T C A G G T A C C T G A T C G A | X-box(HTH)/NPC-H3K4me1-ChIP-Seq(GSE16256)/Homer | 1e-8 | -2.072e+01 | 0.0000 | 494.0 | 1.68% | 368.5 | 1.27% | motif file (matrix) | svg |
| 162 | C T A G C A T G T G C A A C G T A G T C C G T A C A T G T C A G A C G T A C G T G C T A A G T C | Six1(Homeobox)/Myoblast-Six1-ChIP-Chip(GSE20150)/Homer | 1e-8 | -2.063e+01 | 0.0000 | 778.0 | 2.64% | 616.5 | 2.12% | motif file (matrix) | svg |
| 163 | C T A G C T A G A G T C T C A G A C T G A C G T A C G T C T G A | MYB(HTH)/ERMYB-Myb-ChIPSeq(GSE22095)/Homer | 1e-8 | -2.051e+01 | 0.0000 | 8480.0 | 28.77% | 7916.8 | 27.21% | motif file (matrix) | svg |
| 164 | G A C T C T A G A T G C A G T C G T C A T A C G A T G C A T C G | HIC1(Zf)/Treg-ZBTB29-ChIP-Seq(GSE99889)/Homer | 1e-8 | -2.042e+01 | 0.0000 | 10562.0 | 35.83% | 9943.7 | 34.18% | motif file (matrix) | svg |
| 165 | G C A T G C A T C T G A A C G T C T G A A C G T C G T A C G T A C G T A A G T C G T C A G T C A | Foxf1(Forkhead)/Lung-Foxf1-ChIP-Seq(GSE77951)/Homer | 1e-8 | -2.014e+01 | 0.0000 | 3631.0 | 12.32% | 3263.7 | 11.22% | motif file (matrix) | svg |
| 166 | G A C T A G T C C G A T A C T G C T G A T G A C G T A C C G T A A T C G G C A T C T G A C T A G | Bcl11a(Zf)/HSPC-BCL11A-ChIP-Seq(GSE104676)/Homer | 1e-8 | -1.934e+01 | 0.0000 | 2057.0 | 6.98% | 1790.8 | 6.16% | motif file (matrix) | svg |
| 167 | G A C T C T A G C T A G A G T C T G C A A C T G A C G T A C G T C T A G T C A G | AMYB(HTH)/Testes-AMYB-ChIP-Seq(GSE44588)/Homer | 1e-8 | -1.927e+01 | 0.0000 | 6898.0 | 23.40% | 6400.9 | 22.00% | motif file (matrix) | svg |
| 168 | C A T G T A C G T A G C G A T C G A T C A T G C G T A C G A C T T C A G A T G C C G A T A T C G C A G T A C T G G T A C | Zic3(Zf)/mES-Zic3-ChIP-Seq(GSE37889)/Homer | 1e-8 | -1.912e+01 | 0.0000 | 4155.0 | 14.09% | 3770.8 | 12.96% | motif file (matrix) | svg |
| 169 | G C A T A T C G C A T G G T A C G C T A A G T C T C A G T G A C G T C A T G C A | Arnt:Ahr(bHLH)/MCF7-Arnt-ChIP-Seq(Lo\_et\_al.)/Homer | 1e-8 | -1.885e+01 | 0.0000 | 3918.0 | 13.29% | 3547.9 | 12.20% | motif file (matrix) | svg |
| 170 | C T G A A C T G A C T G A G T C A G T C A G C T C T A G T A C G | ZFX(Zf)/mES-Zfx-ChIP-Seq(GSE11431)/Homer | 1e-8 | -1.873e+01 | 0.0000 | 3583.0 | 12.15% | 3230.3 | 11.10% | motif file (matrix) | svg |
| 171 | C T A G T A C G G A T C G T C A T G C A A C G T T G C A G C T A T C G A T G C A | Hoxa9(Homeobox)/ChickenMSG-Hoxa9.Flag-ChIP-Seq(GSE86088)/Homer | 1e-7 | -1.803e+01 | 0.0000 | 10582.0 | 35.90% | 9995.4 | 34.36% | motif file (matrix) | svg |
| 172 | T C A G A G C T A C G T A C G T G T A C G A T C C G T A C T A G C A T G G T C A C G T A T C G A | STAT4(Stat)/CD4-Stat4-ChIP-Seq(GSE22104)/Homer | 1e-7 | -1.786e+01 | 0.0000 | 3701.0 | 12.55% | 3350.0 | 11.52% | motif file (matrix) | svg |
| 173 | G T A C G A T C C A G T A G T C A G T C A G T C T G C A G A T C C T G A A T G C G T C A A C G T | WT1(Zf)/Kidney-WT1-ChIP-Seq(GSE90016)/Homer | 1e-7 | -1.773e+01 | 0.0000 | 3071.0 | 10.42% | 2754.8 | 9.47% | motif file (matrix) | svg |
| 174 | C A T G T G A C C G T A A G T C T A C G G C A T A C T G G T C A A T G C A G T C | bHLHE41(bHLH)/proB-Bhlhe41-ChIP-Seq(GSE93764)/Homer | 1e-7 | -1.757e+01 | 0.0000 | 5604.0 | 19.01% | 5173.8 | 17.78% | motif file (matrix) | svg |
| 175 | A G C T A G T C A G T C A C G T C T A G A C G T A C G T A C G T C G T A A G T C G A T C C G T A | FOXP1(Forkhead)/H9-FOXP1-ChIP-Seq(GSE31006)/Homer | 1e-7 | -1.740e+01 | 0.0000 | 1691.0 | 5.74% | 1463.1 | 5.03% | motif file (matrix) | svg |
| 176 | C A T G A C T G C T A G T C G A T C G A T C G A T C G A T C A G T C A G T C A G T G A C T G A C C G T A A C T G T G C A C G A T A C T G | RBPJ:Ebox(?,bHLH)/Panc1-Rbpj1-ChIP-Seq(GSE47459)/Homer | 1e-7 | -1.729e+01 | 0.0000 | 1349.0 | 4.58% | 1148.9 | 3.95% | motif file (matrix) | svg |
| 177 | A T G C T A C G A G T C G A C T T G C A C T G A G A C T A C G T C T G A T C A G | LHX9(Homeobox)/Hct116-LHX9.V5-ChIP-Seq(GSE116822)/Homer | 1e-7 | -1.710e+01 | 0.0000 | 5668.0 | 19.23% | 5240.6 | 18.01% | motif file (matrix) | svg |
| 178 | T G A C A T G C C G T A A T C G A T G C C A G T C A T G A C T G A G T C G T A C | HEB(bHLH)/mES-Heb-ChIP-Seq(GSE53233)/Homer | 1e-7 | -1.664e+01 | 0.0000 | 8774.0 | 29.76% | 8252.9 | 28.37% | motif file (matrix) | svg |
| 179 | C G A T C T A G T C G A A G C T C G A T C T G A C G T A A G C T A C T G C T A G A T G C G A T C | Hoxb4(Homeobox)/ES-Hoxb4-ChIP-Seq(GSE34014)/Homer | 1e-6 | -1.594e+01 | 0.0000 | 832.0 | 2.82% | 684.2 | 2.35% | motif file (matrix) | svg |
| 180 | A G T C C G A T C T G A C G T A A C G T C A G T T C A G T G A C | Isl1(Homeobox)/Neuron-Isl1-ChIP-Seq(GSE31456)/Homer | 1e-6 | -1.577e+01 | 0.0000 | 7321.0 | 24.83% | 6853.1 | 23.56% | motif file (matrix) | svg |
| 181 | A G T C G T A C A G C T C T A G A G T C C G A T A C T G C G T A A C T G G T C A | Zic(Zf)/Cerebellum-ZIC1.2-ChIP-Seq(GSE60731)/Homer | 1e-6 | -1.523e+01 | 0.0000 | 4838.0 | 16.41% | 4464.7 | 15.35% | motif file (matrix) | svg |
| 182 | C T A G T A C G G A T C G T C A T C G A A C G T T C A G C G T A C G T A C G T A | Hoxd10(Homeobox)/ChickenMSG-Hoxd10.Flag-ChIP-Seq(GSE86088)/Homer | 1e-6 | -1.517e+01 | 0.0000 | 4779.0 | 16.21% | 4408.6 | 15.15% | motif file (matrix) | svg |
| 183 | C T A G A C T G C T A G T C A G T C A G T A C G C T A G A C T G | Maz(Zf)/HepG2-Maz-ChIP-Seq(GSE31477)/Homer | 1e-6 | -1.506e+01 | 0.0000 | 7993.0 | 27.11% | 7515.2 | 25.83% | motif file (matrix) | svg |
| 184 | T C A G G C T A T C A G C A G T T G A C G T C A A G T C A T C G T G C A G T A C C A G T G A T C | Npas4(bHLH)/Neuron-Npas4-ChIP-Seq(GSE127793)/Homer | 1e-6 | -1.501e+01 | 0.0000 | 4409.0 | 14.96% | 4055.1 | 13.94% | motif file (matrix) | svg |
| 185 | G A C T T C G A C G A T T A C G C T A G T A C G A C T G A T G C G T A C G T A C | Zac1(Zf)/Neuro2A-Plagl1-ChIP-Seq(GSE75942)/Homer | 1e-6 | -1.500e+01 | 0.0000 | 9909.0 | 33.61% | 9382.7 | 32.25% | motif file (matrix) | svg |
| 186 | A G T C T A G C G A C T A C G T C T A G A C G T A C G T A C G T C T G A A G T C G C T A G A C T C G T A C T A G A C T G | Foxa3(Forkhead)/Liver-Foxa3-ChIP-Seq(GSE77670)/Homer | 1e-6 | -1.459e+01 | 0.0000 | 1223.0 | 4.15% | 1048.5 | 3.60% | motif file (matrix) | svg |
| 187 | T C A G A G C T G T A C C G T A A C G T C G T A C G T A C G T A G C T A G A C T | Cdx2(Homeobox)/mES-Cdx2-ChIP-Seq(GSE14586)/Homer | 1e-6 | -1.458e+01 | 0.0000 | 2811.0 | 9.54% | 2536.6 | 8.72% | motif file (matrix) | svg |
| 188 | A T G C T C G A T A C G A C G T A T G C A G T C A C G T A G T C A G T C G A T C | Znf263(Zf)/K562-Znf263-ChIP-Seq(GSE31477)/Homer | 1e-6 | -1.438e+01 | 0.0000 | 9071.0 | 30.77% | 8574.2 | 29.47% | motif file (matrix) | svg |
| 189 | C T A G T C G A C G A T C T A G G C A T C A G T C T A G G A T C C G T A G T C A | CEBP:AP1(bZIP)/ThioMac-CEBPb-ChIP-Seq(GSE21512)/Homer | 1e-6 | -1.434e+01 | 0.0000 | 3572.0 | 12.12% | 3262.4 | 11.21% | motif file (matrix) | svg |
| 190 | C A G T T C A G G A T C A C T G A C G T C T A G A C T G A C T G G A C T C T A G | Egr1(Zf)/K562-Egr1-ChIP-Seq(GSE32465)/Homer | 1e-6 | -1.415e+01 | 0.0000 | 3572.0 | 12.12% | 3264.3 | 11.22% | motif file (matrix) | svg |
| 191 | G C T A T C G A C G T A C T A G A G C T G T C A G T C A C G T A A G T C C G T A | FOXA1(Forkhead)/MCF7-FOXA1-ChIP-Seq(GSE26831)/Homer | 1e-6 | -1.408e+01 | 0.0000 | 4064.0 | 13.79% | 3735.7 | 12.84% | motif file (matrix) | svg |
| 192 | C G T A T A G C A G T C C T A G C A G T C T A G C T G A G T A C G C A T T C G A C G T A G C A T A G C T C T A G T C G A | PAX3:FKHR-fusion(Paired,Homeobox)/Rh4-PAX3:FKHR-ChIP-Seq(GSE19063)/Homer | 1e-6 | -1.397e+01 | 0.0000 | 827.0 | 2.81% | 689.7 | 2.37% | motif file (matrix) | svg |
| 193 | C G T A C G T A A G C T G A C T T G C A G T C A A C G T A G C T C T G A T C A G | Lhx3(Homeobox)/Neuron-Lhx3-ChIP-Seq(GSE31456)/Homer | 1e-5 | -1.357e+01 | 0.0000 | 6883.0 | 23.35% | 6459.8 | 22.20% | motif file (matrix) | svg |
| 194 | A G C T T G A C C G A T C G A T C T A G A C G T C A G T C A G T G C T A A G T C | FOXK1(Forkhead)/HEK293-FOXK1-ChIP-Seq(GSE51673)/Homer | 1e-5 | -1.352e+01 | 0.0000 | 3955.0 | 13.42% | 3637.2 | 12.50% | motif file (matrix) | svg |
| 195 | C G A T C T A G A C G T A C G T A C G T C G T A A G C T C G A T A G C T C G T A C T A G T A G C | FoxD3(forkhead)/ZebrafishEmbryo-Foxd3.biotin-ChIP-seq(GSE106676)/Homer | 1e-5 | -1.330e+01 | 0.0000 | 3621.0 | 12.28% | 3320.6 | 11.41% | motif file (matrix) | svg |
| 196 | T A G C G T A C A G T C G T A C C G A T A G T C A G T C A G T C A G T C A G T C C G T A G A T C | Zfp281(Zf)/ES-Zfp281-ChIP-Seq(GSE81042)/Homer | 1e-5 | -1.303e+01 | 0.0000 | 894.0 | 3.03% | 755.9 | 2.60% | motif file (matrix) | svg |
| 197 | T A G C G C T A A C T G C G T A A C G T C G T A C G T A T A C G T C A G T C G A | Gata1(Zf)/K562-GATA1-ChIP-Seq(GSE18829)/Homer | 1e-5 | -1.279e+01 | 0.0000 | 2206.0 | 7.48% | 1980.7 | 6.81% | motif file (matrix) | svg |
| 198 | C G T A G C T A C G A T C T A G A C G T G T C A C G T A C G T A A G T C C G T A T G C A T A C G | FoxL2(Forkhead)/Ovary-FoxL2-ChIP-Seq(GSE60858)/Homer | 1e-5 | -1.276e+01 | 0.0000 | 3262.0 | 11.07% | 2983.3 | 10.25% | motif file (matrix) | svg |
| 199 | A G C T C A T G G C A T G A T C T G C A C T A G G A T C A C G T | Tgif2(Homeobox)/mES-Tgif2-ChIP-Seq(GSE55404)/Homer | 1e-5 | -1.276e+01 | 0.0000 | 12149.0 | 41.21% | 11612.3 | 39.92% | motif file (matrix) | svg |
| 200 | A C T G C A G T A C G T C G T A C G T A A C G T A C T G C T G A | Nkx6.1(Homeobox)/Islet-Nkx6.1-ChIP-Seq(GSE40975)/Homer | 1e-5 | -1.251e+01 | 0.0000 | 10133.0 | 34.37% | 9641.5 | 33.14% | motif file (matrix) | svg |
| 201 | C G A T C T A G A C G T G T C A C G T A C G T A A G T C C G T A | Foxo3(Forkhead)/U2OS-Foxo3-ChIP-Seq(E-MTAB-2701)/Homer | 1e-5 | -1.242e+01 | 0.0000 | 2892.0 | 9.81% | 2634.4 | 9.06% | motif file (matrix) | svg |
| 202 | C T A G T A C G G A T C G T A C G C T A A G C T A G C T G T C A T C G A T A G C | Nanog(Homeobox)/mES-Nanog-ChIP-Seq(GSE11724)/Homer | 1e-5 | -1.225e+01 | 0.0000 | 15126.0 | 51.31% | 14552.1 | 50.02% | motif file (matrix) | svg |
| 203 | C A T G A G T C G A C T C G T A C G A T G C A T G A C T G C A T C G A T C T A G C A T G T G A C | Mef2b(MADS)/HEK293-Mef2b.V5-ChIP-Seq(GSE67450)/Homer | 1e-5 | -1.216e+01 | 0.0000 | 3185.0 | 10.80% | 2916.3 | 10.02% | motif file (matrix) | svg |
| 204 | T A G C G C A T A G T C G A T C A T G C G A C T C T A G A C T G A C T G C T G A A C T G C T A G A G T C T G A C C G A T | GLIS3(Zf)/Thyroid-Glis3.GFP-ChIP-Seq(GSE103297)/Homer | 1e-5 | -1.204e+01 | 0.0000 | 6803.0 | 23.08% | 6404.1 | 22.01% | motif file (matrix) | svg |
| 205 | A T C G A T C G A T G C G A C T T C G A C G T A G C A T A G C T C T G A T A C G | DLX2(Homeobox)/BasalGanglia-Dlx2-ChIP-seq(GSE124936)/Homer | 1e-5 | -1.165e+01 | 0.0000 | 6173.0 | 20.94% | 5799.2 | 19.93% | motif file (matrix) | svg |
| 206 | C A G T C G A T G C A T G C A T G T C A A G C T C A T G C T A G A T G C G T A C | Hoxa11(Homeobox)/ChickenMSG-Hoxa11.Flag-ChIP-Seq(GSE86088)/Homer | 1e-5 | -1.155e+01 | 0.0000 | 9444.0 | 32.04% | 8984.4 | 30.88% | motif file (matrix) | svg |
| 207 | T C A G T C A G G C T A C G T A T A C G G A C T T C A G T C G A C T G A C G T A T A C G G A C T | IRF8(IRF)/BMDM-IRF8-ChIP-Seq(GSE77884)/Homer | 1e-4 | -1.141e+01 | 0.0000 | 1233.0 | 4.18% | 1078.0 | 3.71% | motif file (matrix) | svg |
| 208 | T G A C G C T A T G A C C G T A T C A G G A T C C G T A C A T G C A T G C T A G C T A G C T A G | Unknown-ESC-element(?)/mES-Nanog-ChIP-Seq(GSE11724)/Homer | 1e-4 | -1.131e+01 | 0.0000 | 2930.0 | 9.94% | 2682.6 | 9.22% | motif file (matrix) | svg |
| 209 | C G A T A C G T A C G T A C G T C G T A A G C T C A G T C T A G A T C G A C T G | HOXB13(Homeobox)/ProstateTumor-HOXB13-ChIP-Seq(GSE56288)/Homer | 1e-4 | -1.116e+01 | 0.0000 | 4245.0 | 14.40% | 3944.6 | 13.56% | motif file (matrix) | svg |
| 210 | C A T G G T A C G A C T G C T A C G T A C G T A C G T A G C T A G A C T C T G A T C A G G T A C | Mef2c(MADS)/GM12878-Mef2c-ChIP-Seq(GSE32465)/Homer | 1e-4 | -1.115e+01 | 0.0000 | 1738.0 | 5.90% | 1553.4 | 5.34% | motif file (matrix) | svg |
| 211 | T A C G G A C T A C T G A C T G C T A G A T G C A G T C A G T C A G T C C T G A | ZNF692(Zf)/HEK293-ZNF692.GFP-ChIP-Seq(GSE58341)/Homer | 1e-4 | -1.095e+01 | 0.0000 | 471.0 | 1.60% | 382.4 | 1.31% | motif file (matrix) | svg |
| 212 | T A C G A T C G T A G C G A T C A C T G A C G T A G T C A C G T C T A G A T C G | Smad4(MAD)/ESC-SMAD4-ChIP-Seq(GSE29422)/Homer | 1e-4 | -1.075e+01 | 0.0000 | 5712.0 | 19.38% | 5366.6 | 18.45% | motif file (matrix) | svg |
| 213 | G A T C G C T A C A G T A C G T T A C G A G T C A T G C C T A G A G T C T C G A | Zfp57(Zf)/H1-ZFP57.HA-ChIP-Seq(GSE115387)/Homer | 1e-4 | -1.074e+01 | 0.0000 | 3667.0 | 12.44% | 3394.0 | 11.67% | motif file (matrix) | svg |
| 214 | C A T G A G T C A G C T C G T A C G A T C G A T G C A T G C A T C G A T C T G A C A T G T G A C | Mef2d(MADS)/Retina-Mef2d-ChIP-Seq(GSE61391)/Homer | 1e-4 | -1.037e+01 | 0.0001 | 762.0 | 2.58% | 649.2 | 2.23% | motif file (matrix) | svg |
| 215 | C T A G C G T A G T C A C G T A A G T C G A T C A G C T C T A G C G T A A C G T G T C A G A T C | Six2(Homeobox)/NephronProgenitor-Six2-ChIP-Seq(GSE39837)/Homer | 1e-4 | -1.023e+01 | 0.0001 | 3189.0 | 10.82% | 2942.1 | 10.11% | motif file (matrix) | svg |
| 216 | A G C T A G T C A T G C A G C T A C G T C G T A A C G T A G T C C G A T A T G C | Gata2(Zf)/K562-GATA2-ChIP-Seq(GSE18829)/Homer | 1e-4 | -1.004e+01 | 0.0001 | 2453.0 | 8.32% | 2241.2 | 7.70% | motif file (matrix) | svg |
| 217 | C T G A C T G A C T A G T C G A C G T A A T G C C G T A A C T G C G T A A C G T C T G A C G A T A G C T C G T A A C G T A G T C C G A T T A C G G T C A G C A T | GATA(Zf),IR3/iTreg-Gata3-ChIP-Seq(GSE20898)/Homer | 1e-4 | -1.003e+01 | 0.0001 | 587.0 | 1.99% | 491.3 | 1.69% | motif file (matrix) | svg |
| 218 | A T G C C T G A A T C G T A C G A G T C C G A T T C A G C G A T C T A G A G C T G T C A G T C A C G T A A G T C C G T A T A C G C T G A | Fox:Ebox(Forkhead,bHLH)/Panc1-Foxa2-ChIP-Seq(GSE47459)/Homer | 1e-4 | -1.001e+01 | 0.0001 | 3961.0 | 13.44% | 3686.2 | 12.67% | motif file (matrix) | svg |
| 219 | C A G T G A C T C G T A G C T A G T A C G A T C G T A C G A C T A G C T A C G T T G A C C G T A C A G T A C G T A T G C | ZNF652/HepG2-ZNF652.Flag-ChIP-Seq(Encode)/Homer | 1e-4 | -9.817e+00 | 0.0001 | 882.0 | 2.99% | 763.6 | 2.62% | motif file (matrix) | svg |
| 220 | C T G A A C G T A C G T A C G T A G T C G A C T C G A T C T G A A C T G C G T A C G T A T C G A | STAT5(Stat)/mCD4+-Stat5-ChIP-Seq(GSE12346)/Homer | 1e-4 | -9.631e+00 | 0.0001 | 1143.0 | 3.88% | 1007.4 | 3.46% | motif file (matrix) | svg |
| 221 | C T A G T A G C A G T C G T C A C T G A A C G T C G T A C G T A C G T A G C T A | Hoxd13(Homeobox)/ChickenMSG-Hoxd13.Flag-ChIP-Seq(GSE86088)/Homer | 1e-4 | -9.515e+00 | 0.0001 | 6548.0 | 22.21% | 6197.2 | 21.30% | motif file (matrix) | svg |
| 222 | G T A C G A C T C G T A C T G A T C G A C G T A G C T A C A G T C T G A T A C G | Mef2a(MADS)/HL1-Mef2a.biotin-ChIP-Seq(GSE21529)/Homer | 1e-4 | -9.436e+00 | 0.0002 | 1545.0 | 5.24% | 1386.4 | 4.77% | motif file (matrix) | svg |
| 223 | C A G T T C A G A G C T G A C T A C G T A G T C G A T C G A C T C T G A A C T G G A T C C G T A C T G A A G T C G T A C | Rfx6(HTH)/Min6b1-Rfx6.HA-ChIP-Seq(GSE62844)/Homer | 1e-4 | -9.418e+00 | 0.0002 | 4796.0 | 16.27% | 4500.3 | 15.47% | motif file (matrix) | svg |
| 224 | T G C A A G C T A C G T C T A G G A T C C T A G G A T C G T C A C T G A A G T C | CEBP(bZIP)/ThioMac-CEBPb-ChIP-Seq(GSE21512)/Homer | 1e-3 | -9.206e+00 | 0.0002 | 2614.0 | 8.87% | 2404.6 | 8.27% | motif file (matrix) | svg |
| 225 | A G C T G T C A C G T A A C G T A C G T C T G A T C A G A T G C | Lhx2(Homeobox)/HFSC-Lhx2-ChIP-Seq(GSE48068)/Homer | 1e-3 | -9.188e+00 | 0.0002 | 4367.0 | 14.81% | 4089.4 | 14.06% | motif file (matrix) | svg |
| 226 | A G C T A T G C G A C T G C A T C G T A A G C T G T A C C G A T A T C G A G T C | Gata6(Zf)/HUG1N-GATA6-ChIP-Seq(GSE51936)/Homer | 1e-3 | -9.064e+00 | 0.0002 | 3508.0 | 11.90% | 3264.0 | 11.22% | motif file (matrix) | svg |
| 227 | T A C G C T G A C T A G T C G A C G T A A G T C C T G A A T C G G C A T T A G C G A C T A C T G A C G T A G C T A G T C G A C T | GRE(NR),IR3/A549-GR-ChIP-Seq(GSE32465)/Homer | 1e-3 | -8.956e+00 | 0.0003 | 560.0 | 1.90% | 472.3 | 1.62% | motif file (matrix) | svg |
| 228 | C T A G C T A G C G T A C G T A T A C G C G A T C T A G C T G A C T G A C G T A T A C G G A C T | PU.1:IRF8(ETS:IRF)/pDC-Irf8-ChIP-Seq(GSE66899)/Homer | 1e-3 | -8.944e+00 | 0.0003 | 693.0 | 2.35% | 594.4 | 2.04% | motif file (matrix) | svg |
| 229 | T G C A C G T A G T C A A G C T A G T C G C T A T A G C C G A T C T A G G A T C | Gfi1b(Zf)/HPC7-Gfi1b-ChIP-Seq(GSE22178)/Homer | 1e-3 | -8.691e+00 | 0.0003 | 3073.0 | 10.42% | 2850.0 | 9.80% | motif file (matrix) | svg |
| 230 | T G A C C G A T A C T G A C T G A C T G G A C T A C T G A C G T A C T G A C T G G A T C G A T C | EKLF(Zf)/Erythrocyte-Klf1-ChIP-Seq(GSE20478)/Homer | 1e-3 | -8.556e+00 | 0.0004 | 340.0 | 1.15% | 275.1 | 0.95% | motif file (matrix) | svg |
| 231 | T A G C G A T C G T A C C T G A G C A T C T G A C G T A T G C A C G T A G A T C | Hoxa13(Homeobox)/ChickenMSG-Hoxa13.Flag-ChIP-Seq(GSE86088)/Homer | 1e-3 | -7.928e+00 | 0.0007 | 9667.0 | 32.79% | 9272.0 | 31.87% | motif file (matrix) | svg |
| 232 | A T C G G A T C G C A T C G T A G T C A A C G T A T G C A G T C | CRX(Homeobox)/Retina-Crx-ChIP-Seq(GSE20012)/Homer | 1e-3 | -7.771e+00 | 0.0008 | 7819.0 | 26.52% | 7468.3 | 25.67% | motif file (matrix) | svg |
| 233 | G A C T C G A T T C A G G A T C G A C T A G C T A G C T A G T C G A T C C G T A C T A G C T A G T C G A T C G A C T G A | Bcl6(Zf)/Liver-Bcl6-ChIP-Seq(GSE31578)/Homer | 1e-3 | -7.428e+00 | 0.0011 | 4482.0 | 15.20% | 4228.6 | 14.54% | motif file (matrix) | svg |
| 234 | C G T A A C G T A C T G G T A C C G T A A C G T C G T A C G T A A C G T A C T G A G T C C G T A A C G T C T G A G C A T | OCT:OCT-short(POU,Homeobox)/NPC-OCT6-ChIP-Seq(GSE43916)/Homer | 1e-3 | -7.247e+00 | 0.0013 | 3122.0 | 10.59% | 2918.0 | 10.03% | motif file (matrix) | svg |
| 235 | A T C G A T G C A G T C T A G C G A C T T C G A G C T A G C A T A G C T C T G A | DLX1(Homeobox)/BasalGanglia-Dlx1-ChIP-seq(GSE124936)/Homer | 1e-3 | -7.166e+00 | 0.0014 | 5518.0 | 18.72% | 5238.7 | 18.01% | motif file (matrix) | svg |
| 236 | T A C G A T G C G C T A A C T G C G T A A C G T C G T A C T G A T A C G T C G A | Gata4(Zf)/Heart-Gata4-ChIP-Seq(GSE35151)/Homer | 1e-3 | -7.049e+00 | 0.0016 | 3780.0 | 12.82% | 3555.6 | 12.22% | motif file (matrix) | svg |
| 237 | G C T A A T C G G C T A G A C T G C T A T C G A T A G C T C G A | GATA3(Zf)/iTreg-Gata3-ChIP-Seq(GSE20898)/Homer | 1e-2 | -6.368e+00 | 0.0032 | 5427.0 | 18.41% | 5165.3 | 17.75% | motif file (matrix) | svg |
| 238 | C G T A C T G A C T A G C G T A C G T A A G T C C G T A C A G T G C A T G T C A C G A T A C T G A C G T G C A T G A T C | PGR(NR)/EndoStromal-PGR-ChIP-Seq(GSE69539)/Homer | 1e-2 | -6.340e+00 | 0.0033 | 857.0 | 2.91% | 765.2 | 2.63% | motif file (matrix) | svg |
| 239 | C T G A C T A G T C G A C G T A A T G C C G T A A T C G C G A T T A G C G C A T A T C G G C A T A G C T G A T C G A C T A G C T | ARE(NR)/LNCAP-AR-ChIP-Seq(GSE27824)/Homer | 1e-2 | -6.329e+00 | 0.0033 | 978.0 | 3.32% | 879.5 | 3.02% | motif file (matrix) | svg |
| 240 | A G C T G C A T A C T G A C G T A G T C A C G T C T A G T A C G | Smad3(MAD)/NPC-Smad3-ChIP-Seq(GSE36673)/Homer | 1e-2 | -6.260e+00 | 0.0035 | 9389.0 | 31.85% | 9038.1 | 31.07% | motif file (matrix) | svg |
| 241 | C T A G T C G A C T G A C G T A T A C G G A C T T C A G T C G A G T C A T G C A T A C G A G C T | IRF2(IRF)/Erythroblas-IRF2-ChIP-Seq(GSE36985)/Homer | 1e-2 | -6.241e+00 | 0.0036 | 394.0 | 1.34% | 335.5 | 1.15% | motif file (matrix) | svg |
| 242 | C G T A C A T G A G T C G A C T T G C A C G T A A C G T A C G T C T G A T C A G | Lhx1(Homeobox)/EmbryoCarcinoma-Lhx1-ChIP-Seq(GSE70957)/Homer | 1e-2 | -6.235e+00 | 0.0036 | 4730.0 | 16.05% | 4490.0 | 15.43% | motif file (matrix) | svg |
| 243 | T C G A T C G A T A C G G A T C G T C A G T A C C G A T A G C T G T C A T G C A | Nkx3.1(Homeobox)/LNCaP-Nkx3.1-ChIP-Seq(GSE28264)/Homer | 1e-2 | -6.155e+00 | 0.0038 | 8580.0 | 29.11% | 8248.8 | 28.35% | motif file (matrix) | svg |
| 244 | A T C G G A C T C G A T C G T A C G T A C A G T G A T C G A T C G A T C A G C T | Otx2(Homeobox)/EpiLC-Otx2-ChIP-Seq(GSE56098)/Homer | 1e-2 | -6.068e+00 | 0.0042 | 2617.0 | 8.88% | 2448.2 | 8.42% | motif file (matrix) | svg |
| 245 | A T G C G A C T A C G T C T A G A C G T A C G T A C G T C T G A G A T C G C T A A G C T C G T A | Foxa2(Forkhead)/Liver-Foxa2-ChIP-Seq(GSE25694)/Homer | 1e-2 | -5.980e+00 | 0.0045 | 3079.0 | 10.44% | 2895.2 | 9.95% | motif file (matrix) | svg |
| 246 | T C G A A C T G A C T G C G T A C G T A T C G A A G T C C T G A A T C G G T A C G C A T C A T G | ETS:E-box(ETS,bHLH)/HPC7-Scl-ChIP-Seq(GSE22178)/Homer | 1e-2 | -5.322e+00 | 0.0087 | 360.0 | 1.22% | 309.4 | 1.06% | motif file (matrix) | svg |
| 247 | G T A C C G T A C G T A T A C G G C A T G T A C C G T A C A T G A G T C C G T A C G T A C G A T G C A T G C A T G A C T | MafF(bZIP)/HepG2-MafF-ChIP-Seq(GSE31477)/Homer | 1e-2 | -5.316e+00 | 0.0088 | 1231.0 | 4.18% | 1129.9 | 3.88% | motif file (matrix) | svg |
| 248 | A C G T T A C G G A T C A C T G A C G T C T A G A C T G A C T G G A T C C T A G C A T G C T A G | Egr2(Zf)/Thymocytes-Egr2-ChIP-Seq(GSE34254)/Homer | 1e-2 | -5.271e+00 | 0.0091 | 1056.0 | 3.58% | 963.8 | 3.31% | motif file (matrix) | svg |
| 249 | C G T A A C T G C G T A A C G T C G T A C T G A T C A G T G C A A G C T T G A C | TRPS1(Zf)/MCF7-TRPS1-ChIP-Seq(GSE107013)/Homer | 1e-2 | -5.255e+00 | 0.0092 | 7054.0 | 23.93% | 6777.2 | 23.30% | motif file (matrix) | svg |
| 250 | T G A C C T G A C T A G C T G A C G T A A G T C C T G A A C G T G C A T T A G C G C A T A T C G G A C T G A C T G A T C | GRE(NR),IR3/RAW264.7-GRE-ChIP-Seq(Unpublished)/Homer | 1e-2 | -5.045e+00 | 0.0113 | 886.0 | 3.01% | 804.0 | 2.76% | motif file (matrix) | svg |
| 251 | T C A G C A T G C A T G A C T G A C T G A G C T A C T G A C G T A C T G C A G T A T G C A G T C | KLF10(Zf)/HEK293-KLF10.GFP-ChIP-Seq(GSE58341)/Homer | 1e-2 | -4.971e+00 | 0.0122 | 1723.0 | 5.84% | 1604.2 | 5.51% | motif file (matrix) | svg |
| 252 | T C A G C T A G C T A G T G C A C G A T C G A T C G T A C T A G | GSC(Homeobox)/FrogEmbryos-GSC-ChIP-Seq(DRA000576)/Homer | 1e-2 | -4.907e+00 | 0.0129 | 3898.0 | 13.22% | 3708.6 | 12.75% | motif file (matrix) | svg |
